# Expertise and the “new science” of eugenics

**DOI:** 10.1016/j.socscimed.2025.117829

**Published:** 2025-02-07

**Authors:** Margaret R. Eby

**Affiliations:** Department of Medical Ethics and Health Policy, University of Pennyslvania Perelman School of Medicine, 14th Floor, Blockley Hall, 423 Guardian Dr, Philadelphia, PA, 19104, USA

## Abstract

How are unsettled forms of expertise settled to the advantage of established, but insecure, professional authorities? This research draws on the emergence of eugenics as a “new science” during the first decades of the twentieth century, investigating how medicine came to provide disciplinary authority and organization to eugenic interventions. I do so by analyzing medical publications between 1907 and 1927 to trace physicians’ engagement with eugenic hypotheses, their original research pertaining to eugenics, and the growth of eugenics as an explanatory medical factor. First, I analyze the professional challenge eugenics presented to the rapidly transforming field of medicine and the domain of medical authority. Next, I show first how physicians cast eugenics as both a historical norm and a new science, reinforcing it as part of their domain while protecting their skepticism. Finally, I argue that medicine was able to legitimize unsettled science through a process of “expertise laundering” in which medicine dominated an interdisciplinary exchange of eugenic claims which obscured the unsettled science behind eugenics while bolstering its legitimacy.

“How shall we bring about a ‘wide dissemination of a knowledge of the laws of heredity … ? We shall not be able to reach the public until we are able to influence the education of a body of men whose studies naturally bring them into relation with the subject and who, when united, are numerous enough and powerful enough to sway public opinion. Only one such body of men exists— the medical profession.”

(Galton, 1904)

Between 1907 and 1937, eugenic legislation in the United States increased rapidly. Thirty-three states passed laws allowing for involuntary sterilization on eugenic grounds, while even more initiated public health campaigns to educate their constituents about their responsibility for “better breeding” (Stern, 2016; Nielsen, 2012). Evidence of these new interventions was everywhere: parents entered their infants and children into Better Babies Contests, where they might win prizes based on their performance on series of aptitude tests and physical standards; fairs hosted information booths where inflated statistics about declining American literacy rates and imbalanced reproduction bolstered anti-immigrant rhetoric; newspapers ran headlines warning of growing dependence on state institutions and suggested that eugenics might be the next horoscope through which to find true love (“Eugenics Take the Place of the Constellations: Here is Where Cupid Takes a Scientific Degree in Match-Making” (Smith, 1912)). These projects could be sorted into positive eugenics, which emphasized public education and more of the right kinds of reproduction, and negative eugenics, which included sterilization, institutionalization, and the prevention of marriage among those considered “unfit”.

Although the term ‘eugenics’ was first coined by British physician Francis Galton in 1893, eugenics’ earliest supporters emerged from a variety of disciplines and political projects. The first arguments detailing the dangers of degeneracy and the importance of human breeding came from United States Census agents and women’s suffrage activists (Wine, 1880; Woodhull 1888). Eugenics became an object of medical interest through its necessary role in conducting sterilizations: in an 1892 presidential address to the *Association of Medical Officers of American Institutions for Idiotic and Feeble-Minded Persons*, Dr. Isaac Kerlin asked, “Whose state shall be the first to legitimize oophorectomy (removal of the ovaries) and orchitomia (removal of the testes) for the relief and cure of radical depravity?” With eugenic intervention designated as a surgical intervention, medicine had early access to this new science.

By 1893, castration programs had taken effect under Dr. F. Hoyt Pilcher at the Kansas State asylum. Fourteen years later Indiana became the first state to put eugenic intervention into law. But although surgeons were tasked with practically implementing and supervising eugenic sterilizations in state institutions, eugenic interest had continued to proliferate among professions outside medical authority (Laughlin, 1922). Each structured their support of eugenics as being born of field-centric expertise; botanists claimed Mendel’s experiments with pea plants as the true dawn of eugenics (East, 1923), while feminist reformers argued that as mothers and homemakers, they possessed innate responsibility and knowledge over proper eugenic development (Atkinson, 1910, Eby, 2023). Demographers took the opportunity to call for increasingly detailed population statistics, presenting increased rates of institutionalization as evidence of a growing biological emergency (Sear, 2021). Likewise, sociologists hosted discussions on eugenics as a new branch of social theory and situated themselves as obvious partners in the eugenics project (Galton, 1904). By endorsing eugenics as “the right tool for the job”, these fields attracted capital and recognition as they tried to position themselves as credible experts (Pryma, 2022).

Studies of expertise generally emphasize competition and exclusion as experts and disciplines jockey for ownership. There is no room for collaboration. But the battle for jurisdictional primacy over eugenics unfolded in an unexpected way—despite early contradictions about which disciplinary lineage was the most natural successor to Galton’s eugenics, eugenic expertise became a collective project which afforded its collaborators increased airtime and authority over immediate interventions. This case offers a unique opportunity to examine the processes through which new forms of expertise are legitimized as such and how these news experts accordingly adjusted their new professional boundaries. First, the speed with which eugenics became recognized not only as a new science, but a political and social ethos, indicates the combined power of these experts in constructing and mobilizing interventions. Second, the exchange of pro-eugenics theory and data highlights the importance of inter-field expertise formation and the stakes of various expert bodies in the legitimization of their knowledge.

Here I draw on Eyal’s (2013) sociology of expertise to map the relationships between medicine and other disciplines contributing to the development of eugenics as a science as they negotiate concepts of heredity and control and suggest new strategies for theorizing the exchange of knowledge. The milieu of professions looking for authority and credit through eugenics made implementation of eugenic policy disorganized and wide-ranging. As eugenic interventions were legitimized, so too were their arbiters; this exchange between expert and expertise resulted in a process through which new institutional matrices emerged with the authority to speak on health, reproduction, and population-making. By analyzing leading medical journals between 1907 and 1927, I examine the ways in which medical professionals write about eugenic policy, practice, and practicality, and who they call on to inform or enforce these logics. I find that eugenics became a topic of medical interest during a period of increased instability for the field, when educational reforms and the promise of wider-reaching interventions beyond medicine threatened to destabilize physicians’ authority. I then show how medicine wrangled eugenics under its professional umbrella by defining and contextualizing it as a medical intervention without actually participating in the project of eugenic knowledge creation. Ultimately, I argue that these processes initiated what I call “expertise laundering”, in which the origins of eugenic “expertise” were strategically obscured by filtering them through other disciplines, leaving medicine free to enact eugenic interventions and claim the power that came with them without engaging critically with the logic or science behind them. This research uses the case of eugenics to further develop a theory of expertise that accounts for misalignment in expert task and expertise as well as resulting shifts in expert designation. It also makes the case for a more rigorous analysis of interventions rooted in exclusion, because of, and not despite, dystopian claims of any “new science” of humanity.

## 1. Legitimizing processes

How did medicine legitimize eugenics? In the subsections below, I approach this question both from the standpoint of the new expertise itself and the discipline that adopted it. By evaluating the challenge posed by the new expertise to the developing expert, I develop an analysis of the strategies through which medicine drew on the novelty of eugenics to position itself as its governing body to defray the instability that the growth of eugenics posed to the existing professional order. Finally, I argue that this legitimization was made possible by a process of “expertise laundering” that drew on other disciplines’ authority to bolster claims about eugenics’ authenticity while obscuring the lack of evidence for this new “science”. This theory of expertise laundering draws on the concept of money laundering, the processing of criminal proceeds to disguise their illegal origin. The United States Treasury Department describes money laundering as involving three steps: placement (illegitimate funds are entered into a legitimate financial system), layering (money is moved around to create confusion), and integration (the illegally acquired “dirty money” has moved through enough transaction to appear “clean” (History of Anti-Money Laundering Laws) Likewise, expertise laundering involves the processing of unsettled expertise through various interdisciplinary “transactions” until it appears settled; then disciplines are free to enjoy the new authority these resources bring.

### 1.1. Expertise, experts, and boundary-making

Sociologists have long called for a history of tasks and problems that will account for both jurisdictional struggles over such tasks and the assembly of forms of expertise designed to solve them. While some structure the demarcation of expertise according to professions (Abbott, 2014), others emphasize an expertise-as-network approach. When experts are theorized as a network, arguments about disciplinary power must take into account both the distinction and dynamic interdependence between the monopoly and autonomy experts pursue, as well as the generosity and co-production involved in putting together robust networks of expertise (Eyal, 2013, 899). These networks of expertise require the institutions within them to mutually recognize one another’s expertise as lending something to the shared project. But with eugenics such a “new science”, potential eugenic “experts” had to introduce it as both mutually recognized and beneficial.

As politicians, social reformers, and doctors began to frame failure to take eugenic action as morally reprehensible in the early 1910s, the movement was able to challenge concerns about the ethics of disrupting ‘natural’ (and religious) processes of reproduction (Wilde and Sabrina, 2014). This reframing of eugenics as a moral emergency created a natural historical dovetailing of social and scientific fact (Duster, 2006): eugenics was reframed as the right “tool for the job” for nearly every emerging “challenge” to American society. Eugenics proffered to decrease strain on the state’s social welfare systems, protect against immigrant overpopulation, prevent wars, and, if necessary, ensure the best possible army to win them. The only question remaining was who was to wield the eugenic tools that would enable such a population.

A number of fields claimed eugenics as falling under their purview, with eugenicists themselves encouraging this multidisciplinary approach to eugenics (Laughlin, 1923, 15). Sociologists drawing on Bourdieu’s field theory center the relational nature of knowledge to various fields to highlight the fluctuating value of expertise between disciplines (Panofsky, 2011, 2018; Fligstein and McAdam, 2011; Bourdieu, 1975). These fluctuating values, I argue, meant that medicine could draw on interdisciplinary constructions of eugenics as a “new science” to legitimate its authority without directly engaging with the research behind the valuation of eugenic interventions. The case of eugenics in the first quarter of the 20th century indicates that this process of expertise valuation, and its exchange rate between fields, helped to generate an artificial emergency of legitimization.

These processes of mutual construction between professions and jurisdictions and experts and expertise are effective so long as the problems they seek to solve continue to be recognized under their control. However, when problems move outside their own spheres and appear to endanger society at large, professional routines are criticized, institutions are attacked, elites are threatened and punished, and far-reaching institutional reforms are encouraged (Alexander, 2018). Thus, a problem that escapes a field’s control can likewise challenge field dynamics, disrupting its boundaries, internal hierarchies, and symbolic capital; this, I argue, was the case when eugenics entered medicine’s jurisdiction from the outside.

### 1.2. Theorizing expertise in emerging sciences

Sociologies of medicine in the 20th century point to a social transformation of American medicine through which medicine emerged as a major political and economic player (Starr, 1982). As Americans came to rely on the professional sovereignty of medicine and surrender their private judgment to the institution of medicine, medicalization became a locus of power through which American cultural values about morality and risk were cultivated (Conrad, 1992; Rose, 2001). However, the medical profession also faced a series of professional challenges at the beginning of the 20th century that contributed to its reorganization. In 1910 the Carnegie Institute published Abraham Flexner’s report on American medical schools, which resulted in the closure of one third of operating medical schools (Duffy, 2011). The Flexner Report created more rigid standards for medical education and more rigorous certification exams, unifying medical education and practice under the purview of the American Medical Association (AMA). It also embodied progressive era reformers’ demands for increased social planning and social control in the face of the harmful conditions arising from urbanization and industrialization, endowing newly structured professions with license to autonomously intervene in problem areas (Kunitz, 1974).

The first decade of the 20th century saw enormous gains in favor of medical control over pharmaceuticals as the AMA founded its own regulatory body to discredit the many pills and potions being advertised to the laity, while drug manufacturers increasingly recognized their dependence on doctors’ prescriptions and the medical profession increased its credibility (Starr, 1982, 129). While the expansion of the domain of medicine over the past century can be attributed in part to the prestige the medical profession has accrued and its position as steward of the value of life by performing “miraculous” interventions, it is also the result of medical crusading and entrepreneurship (Conrad and Schneider, 1992). The maneuvering of deviance from “badness” to “sickness” gave medicine even greater control over social life, a control which it wielded throughout the temperance movement, abortion debates, anti-prostitution legislation, and the rise of eugenics.

Through an inductive analysis of these historical accounts, I show how medicine legitimized eugenics as a form of expertise and locate it within its professional domain. I argue that this process had three stages. First, popular support for eugenics and large-scale population improvement interventions infringed on medical authority, encouraging medicine to adapt by widening its scope and maneuvering the challenging expertise under its control. Next, medicine engaged with eugenics conceptually and scientifically by defining and contextualizing eugenics as a settled practice and a progressive, cutting-edge area of expertise. Finally, authors positioned the science of eugenics as incontrovertible by drawing on other disciplines’ claims to eugenic expertise, while denying them jurisdictional authority and strengthening medical claims to this new knowledge. Like the three stages of money laundering— placement, layering, and integration— I argue that these three phases sufficiently obscured eugenic “knowledge” through enough disciplinary transactions that it was transformed into useable “expertise”. This analysis of medicine’s legitimization of eugenics illuminates the strategies by which unsettled forms of expertise are settled to the advantage of established, but insecure, professional authorities.

## 2. Case selection, data & methods

This analysis focuses on a twenty-year period during which twenty-six states passed eugenic sterilization laws, beginning in 1907, when Indiana became the first state to legalize eugenic sterilization, and ending in 1927, when Buck v. Bell was decided by the Supreme Court. I do not suggest that these years fully encapsulate the origins of eugenic logics as they proliferated at the turn of the century or the growth of eugenics through the second world war and beyond. That eugenics has a storied history in national population-making struggles is clear (Stern, 2016; Kevles, 1995; Bashford and Levine, 2010; Largent, 2011). Here I make the case that these twenty years provide some unique insight into the particular growth of support for eugenics as a new science and that these processes by which eugenics came to be recognized as an emergency of medical import, rather than a fringe social reform movement, might add to sociologies of expertise.

Understanding the legitimization of eugenics as a science requires an analysis of the professional discourse surrounding it. This article relies on a mix of primary and secondary sources from medical journals published between 1907 and 1927. These decades cover the early uptake of eugenics as an explanatory factor in individual- and population-level disease, the cultivation of eugenic intervention as a moral duty, and the interaction of eugenics with ideas of scientific progress and nationhood preceding and proceeding the first world war. This data has been collected primarily from American publications for several reasons. First, because the publications examined here continue to hold varying levels of prominence in contemporary medicine, highlighting the legacy of institutions that played, and still play, a significant role in the legitimization of expertise. Second, because American eugenics has been historically de-emphasized in favor of eugenic regimes perpetrated by Nazis during the second World War and the foundations of eugenic theory in genocide perpetrated against indigenous people in the Americas and anti-immigrant policies (Pegoraro, 2015; Sanchez-Rivera, 2023; Black, 2012).

This analysis focuses on three medical journals representing a broad range of orientations to medicine and social intervention. The *New England Journal of Medicine* has been published continuously for 200 years and is currently recognized as the world’s leading medical journal (Brandt, 2012). During the period covered in this paper, the journal was known as the *Boston Medical and Surgical Journal*; it was only in 1928, after a period of financial difficulty, that it was purchased by the Massachusetts Medical Society and reverted to the title by which it is currently known (Campion et al., 2010). The National Medical Association, the first national American organization of African American physicians, began publishing the *Journal of the National Medical Association* in 1908 with the support of the Tuskegee Institute and Booker T. Washington (Morrison and Elizabeth, 2010). The journal continues to be published today with the purpose of addressing medical care disparities of persons of African descent. Finally, the *Medical Review of Reviews* began publication in 1824 and was published under a series of titles at least through 1937 (Ebert, 2012). The *Review* was founded to address the ballooning number of medical journals in the United States; by selecting and reprinting a selection of articles from other medical publications, the *Review* endeavored to keep up with the latest in medicine (F. M. Robinson, 1916). The *Review* is also notable for its investments in public demonstrations and media that represented what it considered to be areas of extreme import for medicine.^1^

These journals, like their profession, represent a period of transition in standards. There was little to interfere with an author’s autonomy - submissions to medical journals were not peer-reviewed until after the second World War, and many journals relied on heavy contributions from their editors to maintain regular publication (Burnham, 1990). Articles described as “original research” might contain analysis of a series of treatment records, census data, a single author’s experience with a certain condition, or simply be a lengthy treatise on the relationship of an illness or treatment to other social conditions. However, they do offer important insights into the public debate and discussion within medicine, as well as demonstrating dominant topics in medical discourse.

A review of these journals resulted in a total of 109 articles (including research articles, reviews, commentaries, and letters) engaging with eugenics. To evaluate the relationships of medicine and eugenics, I developed a series of codes for 1) original research articles pertaining to eugenics, 2) references to inter-field partnerships between medicine and other disciplines in enacting it, and 3) the construction of eugenics as a field, expertise or science. I thus track how eugenics became a legitimized topic of medical expertise on which politicians and social reformers could draw to construct their interventions. In addition to these articles in medical journals, I drew on a wide-ranging set of supporting data. These include original research on eugenics between 1907 and 1927 in the American Journal of Sociology (eighteen articles), the American Journal of Public Health (four articles), accounts of the proceedings of the three International Eugenics Congresses, and contemporary newspaper articles on eugenic conferences and legislation.

## 3. The professional challenge

Eugenics, and the problems it promised to address, entered the scene just as medicine was attempting to strengthen professional boundaries, standardize its training and certification processes, and gain public credibility. In this section I show how eugenic interventions posed new challenges to medical authority and analyze medicine’s response.

Much of this challenge was predicated on the inevitability of a eugenic emergency. Hyperbolic assertions about the impending population crises facing the United States made for popular headlines: from rising rates of immigration to the impacts of war on reproduction, any number of factors were hastening (unwelcome) changes to the population (Salmon, 1913; Leonard, 1914). These threats to the not-so-distant future of the United States rendered interventions attractive. They also lessened the power of medicine’s individually based interventions in improving national welfare. Key medical developments towards the end of the 19th century had been largely in the treatment and prevention of food- and water-borne illnesses, with breakthroughs in the study of bacteria resulting in a transition to the treatment of specific, rather than general, disease. Vaccines against typhoid, tetanus, and rabies emerged at the turn of the century, as did early treatments for syphilis (Starr, 1982, 135). In the early 1910s, however, medical practice began to move its emphasis from drugs to public hygiene. In one 1913 argument for an expansive medical vision beyond pharmaceuticals, a doctor writes that “the term physician is a limiting one, consorts too much with those ideas of ‘physics’ and ‘drugs’. The physician is an ever ready servant in time of trouble, accident and need, but his vision must more and more accommodate itself to the broader horizon of the hygienist” (Goldsbury, 1913). The nation was only as strong as its citizens, making the eugenic demand for the right kinds of bodies a patriotic, as well as professional, duty (Davis, 2016).

The compulsion for a more expansive medical vision was equally met by external pressures for more immediate solutions to pressing population problems. In this 1915 address to the teachers of the Tuskegee Institute Summer School given by Dr. John A. Kenney, personal physician to Booker T. Washington, he references statistics provided in W. Grant Hague’s “The Eugenic Marriage” (Morrison and Elizabeth, 2010):

“It has been stated that 100 years from now the United States will have accomplished what no other race has ever accomplished or it will cease to exist as a nation … in 50 years from now every other child born in this country will either die in infancy or be unfit for self-support, thus being a burden upon society. Here is where the eugenist expects to get in his work. He desires to so enlighten the people and thus change the mode of living so as to bring about a different result.”

(Kenney, 1915)

While medicine grappled with whether eugenics fell within its purview, some physicians and eugenicists argued that declining population quality constituted a national emergency. As Dr. Kenney described here, this is where the eugenicist expected to “get his work”- by offering public, popular immediate interventions. If medicine failed to take up this work, argued some, these movements would be operated at odds with the interests of the profession. Dr. Andrew F. Downing urges his colleagues to engage critically with their role in social progress in a lengthy account of the demands of the 20th century: “There are other social problems involving our profession that are soon to demand an answer. Unless we go forth to meet them with the aggressive and uncompromising spirit of a militant and elevating social force, sex hygiene, eugenics, and birth control will come to us, swaggering with the insolence of popular hysteria and the coarseness of unbridled demagogy” (Downing, 1917).

While eugenics grew in popularity due to its promised solutions to coming emergencies, medicine was facing a crisis of authority. In 1911, Dr. James Ewing, Professor of Pathology at Cornell Medical College, delivered an address at the 64th anniversary of the Academy of Medicine in which he addressed growing public distrust of medicine and argued that demand for immediate, large-scale interventions failed to consider the tremendous innovations in preventive medicine and pathology. The *New York Times* covered the event under the headline, “Public Isn’t Fair to Doctors, He Says— Dr. Ewing at the Academy of Medicine, Attributes its Distrust to Ignorance— Superstition in it, too— Thinks an Inferior Order of Intelligence is a Deterrent in Medical Research”. Ewing acknowledges that while the field was aware of its “defects” as far as medical education, the shortcomings stopped there: “Many influences also in modern times emphasize the incompetence of modern medicine to control the great majority of diseases … The present public distrust, I believe, rests on an inherent defect in the public mind itself.” Ewing goes on to argue that if medicine were no longer serving the public, the only possible reason would be the declining mental quality of individuals entering the profession. While medicine is challenged here because it does not do enough to combat this “great majority” of diseases, Ewing makes a circular argument that this challenge is either coming from those already with defective minds, or because medicine itself has been infiltrated by these same defectives. In this case medicine has taken over the language of eugenics to stave off professional threat and protect its interests. This editorial from the following year, reprinted in the *Medical Review of Reviews* from the *Glasgow Medical Journal*, lays out the professional challenge more plainly-

“The new science of eugenics more and more loudly challenges the interest of physicians and of the laity because it points the way to relief from burdens incident to our civilization, burdens which unless effectively checked tend not to diminish but to increase.”

(Macpherson, 1912)

This new joint interest of physicians and “the laity” would result in increased investment in collaboration, with medicine keen to expand the scope of its expertise over this new science. And because eugenics also threatened a veritable landslide of disasters should preventive measures not be taken, it was only too easy to expand that scope in the interest of a shared moral order. As medicine faced this professional challenge, it undertook the first of the three steps to expertise laundering, by placing the “illegitimate funds” of eugenic logics in a legitimate disciplinary system.

## 4. Defining and contextualizing eugenics

With medicine on the back foot, and its jurisdictional control increasingly precarious, professionals began the process of defining and contextualizing eugenics so that it seemed like a natural extension of the medical profession and a reasonable approach to preventing the pending population emergency. I suggest that medicine was constrained in its response because it lacked sufficient alternatives to eugenics, that is, broad, large-scale interventions that resolved the problems eugenics highlighted as presenting the greatest challenge to powerful interests. Because medicine itself was facing challenges to its authority over the American body, it had more to gain by aligning itself with simplistic eugenic narratives than by challenging them on scientific grounds. As medicine took up the challenge of eugenics, this process occurred both internally (encouraging physicians to take heredity seriously) and externally (presenting eugenics, with medicine in charge, as the most responsible approach to population “emergencies”).

The second step of expertise laundering began here, as eugenics was “layered” in with other disciplinary histories and logics to create confusion around its provenance. The first task for any field hoping to claim eugenics as part of their jurisdictional expertise was to define it as sharing values and properties with other subjects under its authority, and to introduce it as having innate relationships with other topics within that field. American medical journals during this period thus navigated the task of introducing eugenics to their readers in kind: physicians wrote paradoxically of eugenics in this period as both a “new science” and as the most recent iteration of an ongoing strategy for successful population management. These definitions were not distinct, but sometimes posited eugenics as a recently rediscovered science with innately noble qualities rooted in antiquity: “the recent advances which have been made in the knowledge of heredity have awakened interest in the possible artificial improvement of the human race, an idea which dates back to Spartan civilization” (1910 Present Knowledge of the Laws of Heredity, 10). These references to ancient Greek infanticide drew themselves on shoddily constructed classical “expertise” usually drawn from Plutarch’s *Life of Lycurgus*, in which he writes that Spartan infants that were “ill-born and deformed” were abandoned at the foot of Mt. Taÿgetus, “in the conviction that the life of that which nature had not well equipped at the very beginning for health and strength, was of no advantage either to itself or the state” (Plutarch, 1914, 16). This account gave rise to a still prominent theory that the practically-minded Spartans set a precedent for eugenic infanticide as statecraft.^2^

This harkening back to ancient practice was also highlighted in the *Journal of the National Medical Association*: in a 1922 “item of interest” titled “A Reason for Negro Race Pride”, Dr. Sara W. Brown describes the sexual education practices of the Yoruba (from whom she writes a majority of Black Americans descend) as having high standards of individual sexual and racial health. Recounting interactions with a Professor Aggrey of Livingston College, she describes his pride in pointing out the advancement of his people in sex education beyond that of “modern nations”: “I was made to feel that eugenics originated with them” (Brown, 1922). These expressions are surprising because they indicate the prevalence of eugenic logic as extending beyond the promotion and protection of whiteness to an expansive strategy of racial progress, even though the effects of the eugenic project were undoubtedly structured by scientific racism and the goal of white racial dominance. However, the concept of “racial progress” achieved global popularity precisely because it promised a universal improvement in population outcomes and could be constructed as a natural and ancient strategy for achieving them.

Eugenics was also located in medicine’s more recent past, justifying a jurisdictional claim over the practice of contemporary eugenic interventions. Despite Galton being recognized as the modern founder of eugenics, noted a 1916 “Medical Pencillings” section in the *Medical Review of Reviews*, “in an old book called Domestic Medicine, written by Dr. William Buchanan in 1772, we find this: ‘No person who labors under an incurable malady ought to marry. He thereby not only shortens his own life but transmits misery to others’” (“Medical Pencillings” 1916). Eugenics was consistently defined as a science: sometimes as a “new science” (Leonard, 1911), at other times “a science late upon the field” (Goldsbury, 1913); eugenics might be “an applied science” or “only recently recognized as a science” (Kenney, 1915; Taylor, 1912). This balance between ancient standard, naturally arising disciplinary development, and progressive science situated eugenics as a key project of any forward-thinking physician.

This project of linking contemporary eugenics with a formative past also looked to the future, emphasizing the possibilities of eugenic intervention alongside a professional medical imperative to direct them. The then-President of the Pennsylvania State Medical, Dental, and Pharmaceutical Association noted this balance between past and future in his 1923 address:

“Medicine is concerned with the preservation of life and the prevention of disease. To accomplish these ends our profession, the world over, is engaged in research work— co-operative labors endeavoring to transform a progressive art into a fixed science; therefore, preventive medicine is in the ascendency marching boldly on to Eugenics and the “Super-man” via Birth control.^3^”

(Edwards, 1923)

Edwards situates this global work to solidify eugenics as a scientific endeavor as a natural extension of the medical profession, so long as eugenics represents the preservation of life and the prevention of disease. This strategic linking of eugenic ends to the tenets of medicine depicts it as a natural extension of medical expertise and a clear component of medical practice.

As well as positioning eugenics as both a necessary extension of scientific progress and a return to more ancient and natural forms of population control, medical eugenics also posited medicine itself as a driver of progressive interventions. In January of 1911, William J. Robinson M.D., editor of the *Medical Review of Reviews*, wrote an editorial titled “The Medical Profession in the Vanguard of Progress” that “the physician’s broadening interests are naturally beginning to be reflected in our medical periodical literature”. In it he describes recent publications pertaining to eugenics including editorial on “The Birth-Rate and Racial Stability” in the January 7, 1911 *Journal of the American Medical Association*; on the same day, the *Medical Record* published an editorial on the “Decline of the Birth-Rate”; and two days previously, *The Boston Medical and Surgical Journal* an editorial on Eugenics and Divorce. Robinson celebrates this recent uptake of eugenics by the profession, writing that “the world is moving, and let us rejoice that the medical profession is now alive to its opportunities and its responsibilities, and is the very vanguard of progress” (W. J. Robinson, 1911). The de-linking of science from the origins of eugenics while situating contemporary medicine as a natural driver of eugenic progress initiated the cycle of obscuring evidence for eugenics while claiming authority over it.

## 5. A collaborative expertise

The third piece of this process of legitimization is the organization of a collaborative expertise. Throughout these processes of first defending medicine against the professional challenge posed by eugenics, and later the defining and contextualizing eugenics as an extension of medical expertise, contributors to these medical journals drew on interdisciplinary accounts of this new science to emphasize its credibility. By mutually affirming the possibility of eugenic interventions, medicine engaged other disciplines like statistics and sociology in a process of expertise laundering that obscured the unsettled science behind eugenics while bolstering the legitimacy of an intervention that would assure medical authority. Eugenics had become a “clean” science after sufficient transactions integrated it into existing disciplinary frameworks.

Medicine, facing a credibility crisis as eugenics offers more immediate and expansive solutions to a perceived population emergency, saw the incorporation of eugenic expertise to expand its professional authority and benefit from the broad support eugenics received socially and politically. In rendering eugenics a medical topic, authority of treatment and control of the human body was centralized under medical authority. Physicians thus introduced eugenics in their professional writing as a natural extension of medical authority and normalized it as an intervention historically conducted by respected civilizations. Beyond this, eugenic intervention was framed as a medical responsibility-to avoid acting on such clear evidence would make medicine complicit in the inevitable population disaster. But what evidence was medicine relying on when justifying its commitment to the eugenic project? And how could such a “new science” justify the reforms it needed to operate? Despite sweeping claims about the efficacy of eugenics, the medical publications analyzed here do not draw on original data to demonstrate either the eugenic emergency or the efficacy of eugenic interventions. Instead, they rely on data passed through a series of “transactions” in order to legitimize them. Arguments that at their outset were clear political projects passed through various journals and conferences to emerge as the result of collaborative, empirical research. This laundering process drew on other disciplines’ constructions of eugenics and combined them with “common sense” medical logic, in combination with narratives of medical authority over the body and reproduction, to present the unsettled “science” of eugenics as actionable and legitimate. This worked by obscuring the origins of eugenic “expertise” to deny other disciplines authority over eugenics and avoid challenges to the primacy of eugenic interventions. Eugenics was thus laundered through other disciplines until it arrived, settled and useable, as a medical intervention. Other disciplines were willing to participate in this laundering process because their expertise alone lacked the institutional strength to transform eugenic expertise into professional authority. Medicine assembled these contributions from statistics, sociology, and demography into a cohesive set of eugenic knowledges that supported demand for medical intervention. And by lending their support to medicine, each discipline participating in expertise laundering elevated their status through attachment to the eugenic project.

One example of such a laundering relationship was between medicine and sociology. Eleven months after the editor of the *Medical Review of Reviews* rejoiced at the increase in medical articles about eugenics, the journal announced that it would install as one of its main features a monthly “Department of Medical Sociology, devoted to matters bearing on the economic bases of disease, preventive medicine in its larger social relations, and to the study of the agencies under social control that may improve or impair the physical and mental qualities of future generations.” “The pill-and-potion doctor is not the modern doctor,” read the announcement of this new section. “The sphere of the medical man has widened.”

This strategy of relocating extra-medical research on eugenics to within medical spaces, under medical authority, was the final step in ensuring that medicine became the main arbiter of eugenic interventions. This process of expertise laundering, through which the origins of eugenic “expertise” were obscured by relying on other disciplines to provide the professional credibility behind the research, meant that the authority of this newly legitimate science flowed back to medicine and bolstered its reputation. This process had begun even as medicine defended itself against the professional challenge and defined and contextualized eugenics within medicine. In early attempts to define and contextualize eugenics as a reasonable and medical intervention, suggestions were made that the physician become more like any number of professionals whose work made them experts in heredity (Goldsbury, 1913, 643; Taylor, 1912, 833); however, these suggestions never went so far as to grant jurisdictional authority over eugenics to these professions. Instead, their role was to formally situate the application of their suggested interventions under medical control. Here Southard claims that in comparison to other social issues, like alcoholism and venereal disease, feeblemindedness moves to the forefront because it is a problem that can actually be solved through reasonable interventions:

“What chance has the problem of the feeble-minded with countless other problems of civilization? I should suppose, however, that statistics as a whole and the special kind of analytical statistics known as eugenics has gone far enough to prove that the social philosophy of prevention has wide scope … statistics tend to indicate that the problem is rather a medical than an economic one.”

(Southard, 1914)

These interventions, Southard suggests, must come from medicine-not because medicine itself is demanding control of the new science of eugenics, but because the field of statistics- and eugenics itself, called here a special kind of analytical statistics-has proven that the problem is medical, rather than economic. As Southard was at that time an assistant professor of psychology at Harvard University and Bullard Professor of Neuropathology at Harvard Medical School, as well as a pathologist for the Massachusetts Commission on Mental Diseases, it was in his professional interest that eugenic interventions fall under medical, rather than economic, control.

But as medicine cemented its hold on eugenics as a form of expertise, it continued to defer questions about the follow-through of suggested eugenic interventions to other disciplines: in an address to the Cape May Medical Society in 1922, Dr. Alfred M. Gordon reckons with the divisions of labor in the implementation of eugenics: “The problem of race betterment embraces the two fundamental elements of eugenics, namely the knowledge of the laws of heredity and sterilization of the mentally unfit … The questions naturally arise. How and by whom should this education be carried out?” But, as Gordon goes on to say, these are not medical problems, but rather “practical questions of sociologic dimensions”. This element of practical eugenics is beyond medical purview (Gordon, 1923). Here Gordon delineates the professional tasks of medicine as they pertain to medicine— particularly in cases of negative eugenics, the structuring of eugenics policy, and the application of the interventions it entailed— and the professional tasks that might be jettisoned to other professions that would deal with the dissemination of positive eugenic messages. With questions of educational application and feasibility successfully deferred, the science of eugenics was kept firmly within the domain of medicine.

Although such discussion and engagement relied on extra-medical knowledge and data, these were construed as extensions of medical expertise that expanded, rather than infringed upon, medical turf. In a *Medical Review of Reviews* article on social diseases and public enlightenment, Dr. Irving Wilson Voorhees marked the “growing tendency” to study what had been previously thought of as strictly medical problems from a social and eugenic standpoint. He credited the medical profession with encouraging the study of prevention of disease, which was now “beginning to bear fruit.” He specifically names local societies disseminating information on sexually transmitted diseases and the field of sociology with doing the most significant amount of work in this direction, but adds that still more must be done to pursue and eradicate anti-eugenic “social diseases” (Voorhees, 1913). Voorhees’ construction of non-medical eugenics work as the result of medical incitement positions medicine at the head of the eugenic body.

These collaborations occurred during a particularly troubling time for many scientists, as the effects of the first World War (at that time the deadliest conflict in human history) became clear and the lofty ideals for a century of scientific collaboration and progress were dashed. “The loss of millions of useful lives has impressed the world anew with the value of life. And now the scientific world has roused itself,” said Dr. Preston Edwards in a 1923 address. “The great research laboratories of France, England, Germany, and the United States have called to their assistance the chemist, biologist, and physicist, to aid in solving some of our problem of life preservation, knowing that life and health demand scientific cooperation.” (Edwards, 1923) Scientific collaboration itself was a strategy for continued peace, and the eugenics ends to which it worked promised more permanent solutions: as a Dr. A.L. Goldwater of Central Park West wrote, voluntary sterilization, and the resulting limitation in the number of offspring among the middle, and especially among the poorer, classes, would solve economic and social problems that it would “end wars, do away with child labor, and incidentally solve the 8-h day problem” (Goldwater, 1917). Cooperation and collaboration with other sciences was to mutual social and economic benefit and would ultimately secure a future free of the struggles of the early decades of the 20th century.

## 6. Conclusion

This study traces the processes through which eugenics became designated as an area of medical expertise. In American medical journals, eugenics was introduced as a “new science” without a scientist; surgical sterilizations brought it under the purview of medicine without further examination of its ultimate goals. The framing of eugenics as a response to a pending population emergency immediate action and put medicine on the back foot. As the sources I examine here show, demand for a “physician of the future” pushed medicine to define and contextualize eugenics as both a path to human progress and a natural extension of medical expertise. Where medicine lacked the data and theory to support eugenic intervention, it facilitated a cycle of expertise laundering as medicine and the social sciences traded data and logics to cement the legitimacy of eugenics while affirming their expert domains.

Despite eugenics’ emergence as a key driving ideology behind social and scientific interventions over just a few decades, there was scant evidence to justify its far-reaching reforms. Modern accounts of eugenics are often quick to reduce it to a pseudoscience, minimizing its influence on the disciplines and institutions that drove its success and the legacies of exclusion and control it left behind. Future research on the relationships of expert jurisdictional struggles and the legitimization of new forms of expertise should examine the role of outside forces (private industry, social sciences, state and political agencies) on funding or encouraging the adoption of new interventions in the face of medical reluctance. As I demonstrate here, the construction of eugenics as a solution to impending emergency was the result of targeted social and political action around immigration anxieties and manufactured popular science. Narratives around productive and successful human life cannot be delinked from economic cycles of production and consumption or racist, xenophobic, and ableist population fears.

Eugenics was one avenue through which ideals of citizenship were used to control access to property, marriage, and reproduction (Ben-Moshe et al., 2014; Davis, 2016). Although the term has fallen out of favor, the logics that enable eugenic proliferation are ever-present in conflicts over changing demographics and the ideal body. New population “emergencies” are gaining in popularity, from the “Great Replacement” theory, which argues that white populations are being demographically displaced by non-white populations in Europe and the United States (Molteni, 2022; Misra, 2022), to global pro-natalist movements to increase certain populations considered to be in decline (Cook et al., 2023; Lee, 2024). Meanwhile, as genetics and genomics research offer expansive interventions at all stages of life, challenging the boundaries of ethical innovation, it is increasingly vital to critically engage with ongoing legacies of eugenics in science, technology, and medicine (Rembis, 2009; Comfort, 2018; Benjamin, 2016, Obasogie and Darnovsky, 2018; Mukherjee et al., 2022).

With this research I add to the literature on expertise by demonstrating the relationships between experts, their expert jurisdictions, and the expertise laundering that enforces their boundaries while affirming their ownership of the expert tasks at hand. I also aim to challenge histories of medicine and the social sciences which fail to adequately account for the processes through which harmful expertise is generated. As old population fears emerge alongside new reproductive possibilities, a historical perspective on the mechanisms that drive the adoption of such new sciences offers important insight into potential outcomes.

## Data Availability

Data will be made available on request.

## Footnotes

1 These included the production of plays on public morality (including an American run of 1901 French play “Les Avariés” (“Damaged Goods”), about the effects of syphilis on a marriage) and a pro-eugenics demonstration in which “several men from the bottom strata of life” were hired to march through New York City street bearing signs such as, “I am a burden to myself and the State. Should I be allowed to procreate?” and “Would the prisons and asylums be filled if my kind had no children?” (F. M. Robinson, 1916).

2 However, there is little evidence beyond Plutarch for this tradition, and more recent scholarship has situated the popularity of this claim as yet another attempt to normalize and contextualize standards of disability (Sneed, 2021).

3 “Birth Control” in this case refers to any intervention designed to control reproduction, on an individual or population scale. Pharmaceutical birth control for women’s private use was not yet developed.
